# Supplementary figures and images for: The role of biotic factors during plant establishment in novel communities assessed with an agent-based simulation model
Source: PeerJ. 2018 Aug 8;6:e5342. doi: 10.7717/peerj.5342 (PMC6087422; doi:10.7717/peerj.5342)

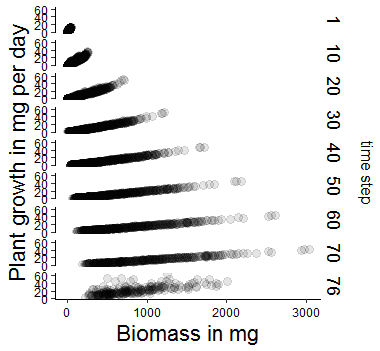

Supplement: Figure S1 — Each point cloud represents one daily time step in the model as indicated on the righthand side. Plant biomass has a strong influence on plant growth in the model. [file peerj-06-5342-s001.png]
