# Supplementary material for: The role of biotic factors during plant establishment in novel communities assessed with an agent-based simulation model
Source: PeerJ. 2018 Aug 8;6:e5342. doi: 10.7717/peerj.5342 (PMC6087422; doi:10.7717/peerj.5342)
Supplement: Supplemental Information 1 — Motivation, methods, results, conclusions and figures related to alternative simulations with proportional herbivory. This means, herbivores consume relative amounts of plant biomass instead of absolute amounts in the main simulations. [file peerj-06-5342-s002.pdf]

Supplemental Material S1: Alternative herbivory simulations

Attached to Radny & Meyer: The role of biotic factors during plant establishment in novel communities assessed with an agent-based simulation model

Motivation

In the standard version of our simulation model, herbivores consume an absolute amount of biomass per plant of 7.4 - 7.6 mg. This might lead to relatively higher losses in terms of percentage foliage of small plants compared to lager plants. To investigate whether and how this affects model results, we adjusted the analysis for this effect by implementing herbivore consumption as relative amounts of plant biomass in an alternative model version.

Methods

In the alternative model version, each herbivore consumes between 1.2% and 4.5% of the total current biomass of a plant that it successfully attacked. The mean of these percentages corresponds to the percentage of the mean biomass of all plants in a typical simulation that is represented by 7.5 mg.

NetLogo Code snippet from the standard version:

```
set biomass_now biomass_now - ((7.5 * (1 + random-float 0.1)) * count herbivores-here)
```

NetLogo Code snippet from the alternative version:

```
set biomass_now biomass_now * ((0.988 - (random-float 0.033 * count herbivores-here)))
```

The alternative model version was run and analyzed in the same way as the standard model version (see section *Scenario analysis* in main text). We plotted graphs corresponding to Figures 4-7 in the main text.

Results

We found qualitatively similar results for standard and alternative model versions (**Fig. 4-Alternative** – **Fig. 7-Alternative**). The main difference was that the effect of high competitiveness traits on survival was much less pronounced in the alternative than in the standard version. Furthermore, the effect of high defense traits on survival was more visible (or at all detectable) in the alternative than in the standard model version.

Conclusions

We conclude that the qualitative results of our model are robust towards different herbivory implementations. The quantitative differences indicate, though, that it would be worthwhile to explore a greater range and resolution of herbivory implementations in the future. These will require more data for parameterization than are currently available. Thus, concerted empirical-modelling efforts are called for.

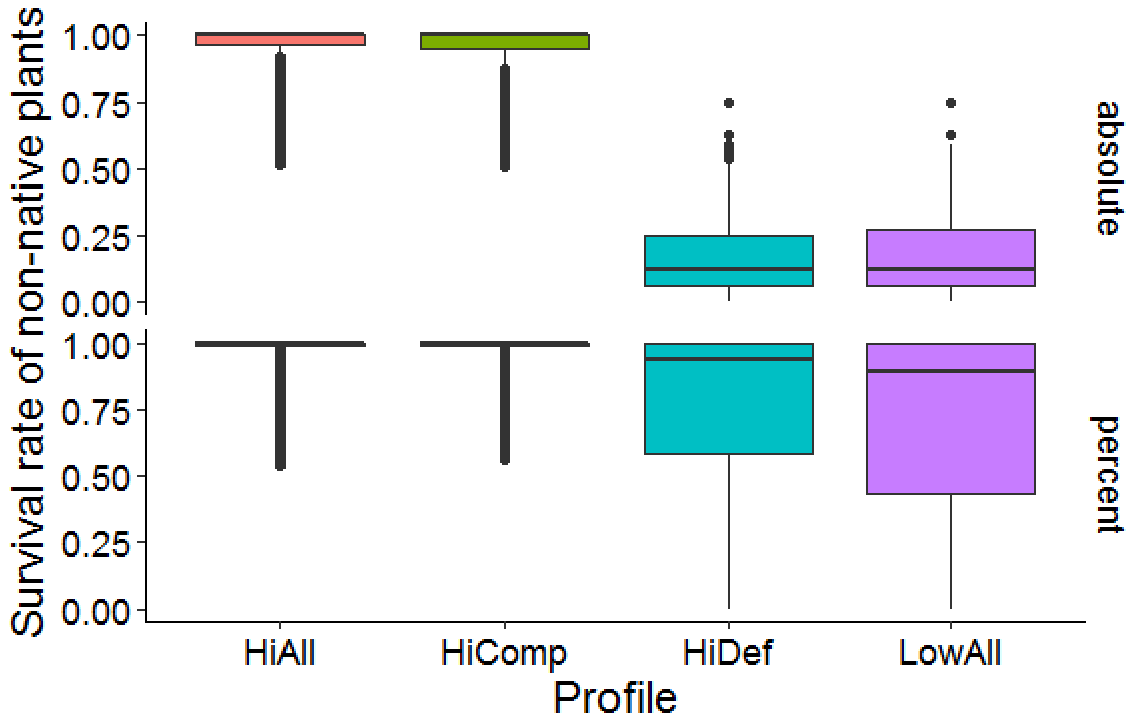

Fig. 4-Alternative. Survival rates of non-native plants with different trait profiles for the standard (absolute) and alternative (percent) model version. The trait profiles are: high competitive and high defensive (HiAll), high competitive and low defensive (HiComp), low competitive and high defensive (HiDef), and low competitive and low defensive (LowAll). Values were averaged over all scenarios.

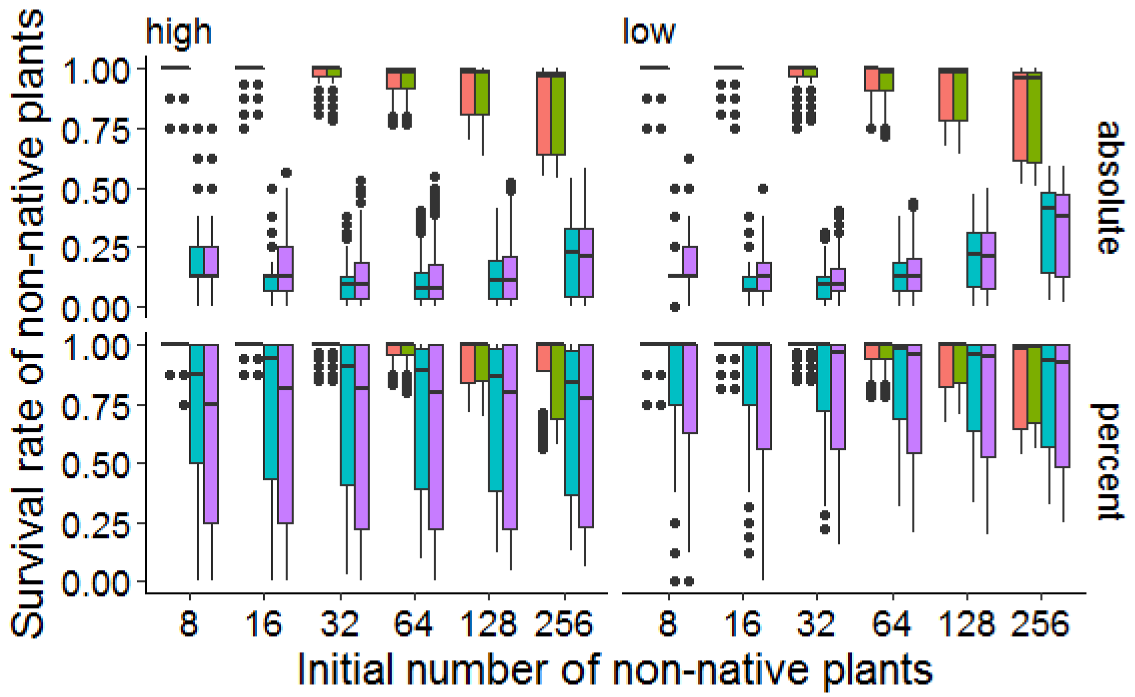

Figure 5-Alternative: Survival rates of non-native plants under different invasion levels in high density (left panels) and low density (right panels) native communities for the standard (absolute) and alternative (percent) model version. Invasion level corresponds to initial population sizes of 8, 16, 32, 64, 128 and 256 individuals of the non-native species. Non-natives are split into the following trait profiles (in each block from left to right): high competitive and high defensive (HiAll, orange), high competitive and low defensive (HiComp, green), low competitive and high defensive (HiDef, blue), and low competitive and low defensive (LowAll, purple).

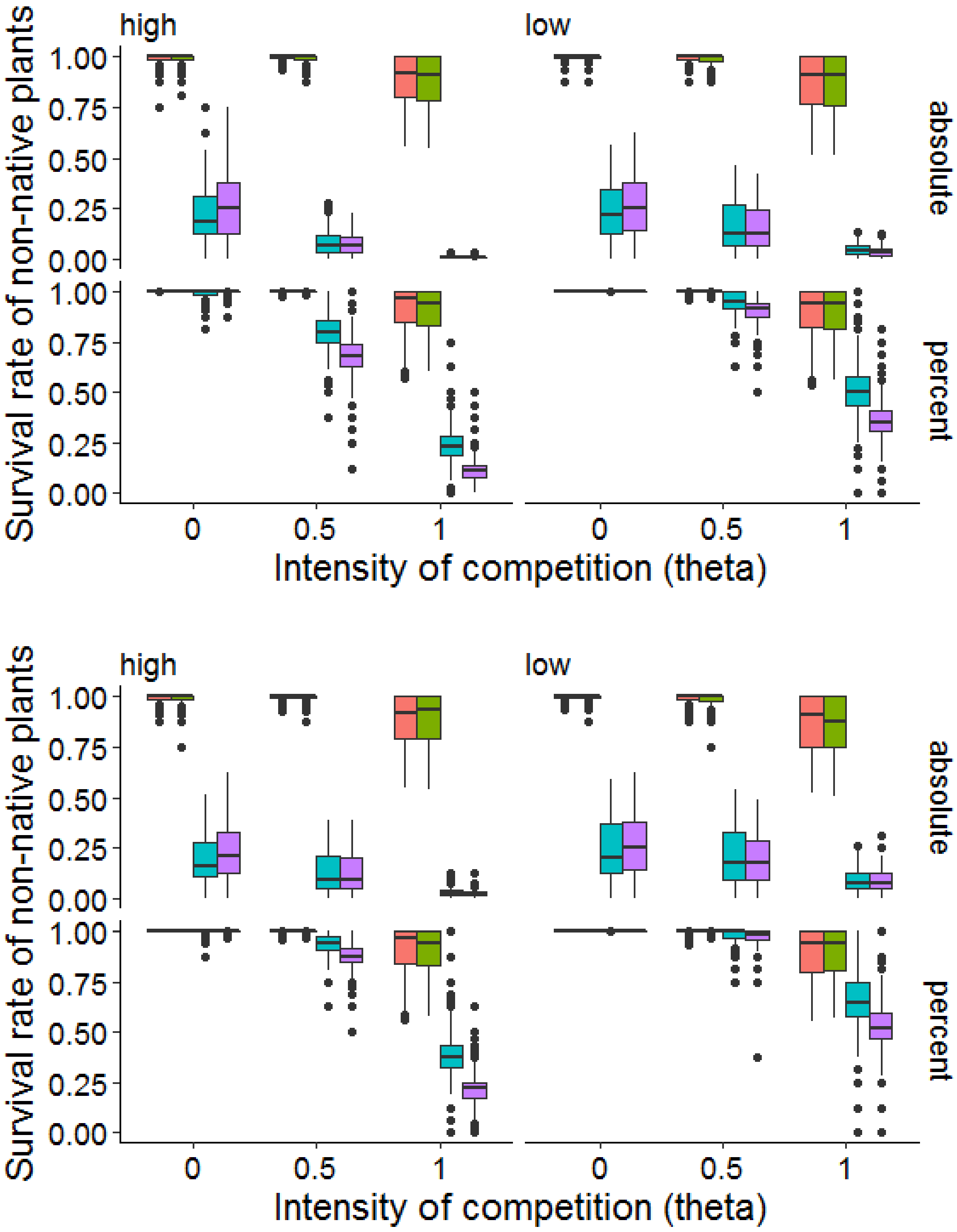

Figure 6-Alternative: Effect of the intensity of competition Θ on survival rates of non-native plants in different configurations of the resident community for the standard (absolute) and alternative (percent) model version. Left panels show high density of natives, right panels show low density of natives. Note that different initial numbers of non-natives are not separated in this figure. The upper four panels show an even mixture of natives in the initial community, the lower four panels show a mixture based on literature values of seed bank sizes. With Θ = 0, resources are shared among competitors regardless of their biomass, with Θ = 1, resources are shared proportionally to the biomass of the individual competitors. Θ = 0.5 reflects an intermediate stage. Non-natives are split into the following trait profiles (in each block from left to right): high competitive and high defensive (HiAll, orange), high competitive and low defensive (HiComp, green), low competitive and high defensive (HiDef, blue), and low competitive and low defensive (LowAll, purple).

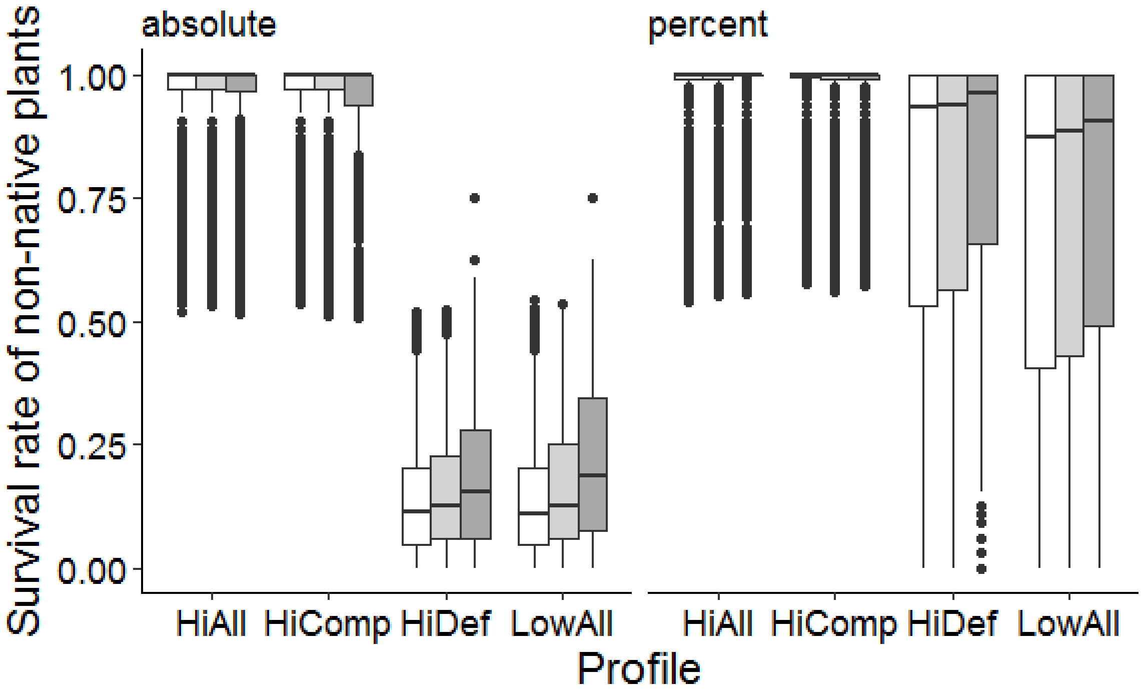

Figure 7-Alternative: Survival rate of non-native species at different herbivore densities for the standard (absolute) and alternative (percent) model version. Herbivore densities: no herbivores as control scenario (white bars), 3.2 herbivores per m2 (light grey bars), and 18.75 herbivores per m2 (dark grey bars). Non-natives are split into the following trait profiles: high competitive and high defensive (HiAll), high competitive and low defensive (HiComp), low competitive and high defensive (HiDef), and low competitive and low defensive (LowAll).
